# Nanopore sequencing and full genome de novo assembly of human cytomegalovirus TB40/E reveals clonal diversity and structural variations

**DOI:** 10.1186/s12864-018-4949-6

**Published:** 2018-08-02

**Authors:** Timokratis Karamitros, Bonnie van Wilgenburg, Mark Wills, Paul Klenerman, Gkikas Magiorkinis

**Affiliations:** 1 Department of Zoology, University of Oxford, Oxford, United Kingdom; 2 Public Health Laboratories, Department of Microbiology, Hellenic Pasteur Institute, 127 Vas Sofias Ave, 11527 Athens, Greece; 3 Nuffield Department of Clinical Medicine, University of Oxford, Oxford, United Kingdom; 4 Department of Medicine, University of Cambridge, Cambridge, United Kingdom; 5 NIHR Biomedical Research Centre, Oxford, United Kingdom; 6 Department of Hygiene, Epidemiology and Medical Statistics, Medical School, National and Kapodistrian University of Athens, M. Asias 75 str., 11527 Athens, Greece

**Keywords:** Human cytomegalovirus, Nanopore, MinION, de novo assembly, Recombination, Mutation, Variable number tandem repeats, Quasi-species

## Abstract

**Background:**

Human cytomegalovirus (HCMV) has a double-stranded DNA genome of approximately 235 Kbp that is structurally complex including extended GC-rich repeated regions. Genomic recombination events are frequent in HCMV cultures but have also been observed in vivo. Thus, the assembly of HCMV whole genomes from technologies producing shorter than 500 bp sequences is technically challenging. Here we improved the reconstruction of HCMV full genomes by means of a hybrid, de novo genome-assembly bioinformatics pipeline upon data generated from the recently released MinION MkI B sequencer from Oxford Nanopore Technologies.

**Results:**

The MinION run of the HCMV (strain TB40/E) library resulted in ~ 47,000 reads from a single R9 flowcell and in ~ 100× average read depth across the virus genome. We developed a novel, self-correcting bioinformatics algorithm to assemble the pooled HCMV genomes in three stages. In the first stage of the bioinformatics algorithm, long contigs (N50 = 21,892) of lower accuracy were reconstructed. In the second stage, short contigs (N50 = 5686) of higher accuracy were assembled, while in the final stage the high quality contigs served as template for the correction of the longer contigs resulting in a high-accuracy, full genome assembly (N50 = 41,056). We were able to reconstruct a single representative haplotype without employing any scaffolding steps. The majority (98.8%) of the genomic features from the reference strain were accurately annotated on this full genome construct. Our method also allowed the detection of multiple alternative sub-genomic fragments and non-canonical structures suggesting rearrangement events between the unique (UL /US) and the repeated (T/IRL/S) genomic regions.

**Conclusions:**

Third generation high-throughput sequencing technologies can accurately reconstruct full-length HCMV genomes including their low-complexity and highly repetitive regions. Full-length HCMV genomes could prove crucial in understanding the genetic determinants and viral evolution underpinning drug resistance, virulence and pathogenesis.

## Background

Human cytomegalovirus (HCMV) is a betaherpesvirus, with the largest known genome of all human herpesviruses. HCMV is pathogenic during both primary infections and reactivations, while the disease impact is more severe in individuals with acquired or developmental deficits in innate and adaptive immunity [1]. The 235 Kbp-long double-stranded viral genome is partitioned in two major segments, the Unique Long (UL) and the Unique Short (US) both flanked by terminal -T- and internal -I- repeated sequences (TLR/ILR, TSR/ISR) [2]. These two genomic segments may invert with respect to each other resulting in four genomic isomers, which can be present in equal concentrations [2] suggesting that HCMV is characterized by extended structural genomic plasticity. Other major recombination events and genomic rearrangements have been observed in vitro and in vivo [3], for example the laboratory strain AD169 has lost a 15 kbp fragment – including 19 ORFs – from the UL/b’ region [4], compared to the reference sequence Merlin (NC_006273) [5]. Crucially, wild-type HCMV strains cannot be readily cultured, but laboratory strains AD169 and Towne can replicate efficiently in fibroblasts, which has made them the most important strains in HCMV research for decades. Strains TB40/E and TB40/F were derived from a bone marrow transplant recipient by passaging in endothelial cells and fibroblasts, respectively [6]. TB40E is the only endotheliotropic strain, which also infects monocytes and has the unique capability to impair their chemokine-driven migration, by down-regulating surface chemokine receptors [7]. Currently, only 3 full genome sequences of TB40E strain are publicly available, and one of them is cloned in a BAC vector [8, 9]. Clones “Lisa” and “Bart” were originally plaque-picked from TB40/E cultures to isolate clones that could or could not evade Natural Killer (NK) cells function [9].

Back in 1990 the first full genome sequence of the highly-passaged strain AD169 was published based on overlapping PCR amplified fragments, cloning and traditional Sanger sequencing [10]. Since then, multiple efforts have been made to isolate full genomes from clinical and other low or moderately passaged laboratory strains [11]. In the era of high throughput sequencing (HTS), dedicated library preparation protocols have been developed to enhance the full genome sequencing of HCMV, based on target enrichment [12], host DNA depletion and whole genome amplification [13] or on multiple amplicon deep sequencing [5]. All of these methods involve some sort of PCR amplification during the library preparation, which has been clearly shown to introduce artificial recombinants [14].

In 2013, Oxford Nanopore Technologies (ONT) announced a new, long-read, third-generation sequencing platform based on nanopore sequencing, the MinION, through an early access program (MAP). This USB-interfaced lighter-sized sequencer is commercially available since 2015 and is able to produce up to 10 Gbases of data from a single flowcell, which comprises an array of 512 nanopores. Whilst the longest known read is 950 Kb, the accuracy of the first version of the sequencer did not exceed 72% [15]. However, the latest flowcells, which employ recurrent neural networks (R9 RNN), have been improved dramatically and now provide more than 92% accuracy for the double stranded (2D) reads. The improved accuracy and the incomparable portability of this tiny, third generation sequencer makes it very attractive for point-of-care applications. MinION has been used in combination with other sequencing platforms that deliver shorter reads of higher accuracy, to improve the hybrid de novo assembly of genomic regions that are difficult to be resolved [16] and of Human Herpes Virus 1 (HHV1) genome [17].

In the present study we have both reconstructed the full genome of HCMV strain TB40E and captured quasi-species diversity of HCMV in culture. We were able to produce a single genomic contig without external scaffolding assistance [13], thus showing for the first time that extra-long MinION reads facilitated the resolution of technically challenging assemblies by passing through repetitive elements which disrupt the assembly of contigs when generated by traditional short-read sequences. We also describe genomic rearrangements in sub-genomic fragments and non-canonical structures that imply the existence of non-canonical HCMV genomes in our cultures.

## Methods

### Cell culture isolation and virus preparation

Human foreskin fibroblasts (HFF), gift from the Sir William Dunn School cell bank, were cultured in Dulbecco’s Modified Eagle Medium (DMEM) (Gibco) containing 10% fetal calf serum (FCS). All cell cultures were tested negative by 4,6-diamidino-2-phenylindole (DAPI) staining for mycoplasmas. HCMV strain TB40/E (the original virus isolate was kindly provided by Prof. C. Singzer, University Hospital Ulm [6, 8]) was prepared by infecting HFF at an m.o.i. of 0.1 p.f.u. per cell. Once > 90% of the cells were showing signs of cytopathic effect (cpe), supernatants of infected cultures were harvested every two days until cells were observed to lose their adherence to the plastic flask surface. Supernatants were stored at − 80 C after removal of cell debris by centrifugation for 10 min at 2800 g. At a later time, the virus preparations were thawed, pooled and further concentrated through ultracentrifugation (12,000 rev for 2 h) using a Type 19 rotor and Beckman Ultracentrifuge. The resultant pellets were washed over gently with sterile Phosphate Buffer Saline (PBS) and finally re-suspended and combined into 1 mL DMEM. The infectious titre in HCMV preparations was determined by plaque assay.

### MinION sequencing

DNA was extracted using the “PureLink Viral RNA/DNA Mini Kit” (Invitrogen) and was quantified with the “Quant-iT PicoGreen dsDNA Assay Kit” (Invitrogen). The DNA purity was evaluated using a NanoDrop™ spectrophotometer and a sample with A260/280 and A260/230 ratios values greater than 1.8 was chosen. Approximately 500 ng of extracted nucleic acid was processed for sequencing and MinION sequencing libraries were prepared using the ONT “Rapid Sequencing Kit”. The kit makes use of a specially formulated transposase to fragment the DNA in a relatively larger size than in other platforms, while it attaches the sequencing adapters at the same time. The library was finally loaded on a R9-RNN flowcell attached on a MinION MkI B sequencer for a 48 h run with real-time data processing.

### Bioinformatics

MinION basecalling was performed on the cloud using the “Metrichor” agent (provided by ONT). We converted the *.fast5* reads to *.fasta* files using the poRe package for R programming language. The alignment of the reads was performed with *LAST* setting the alignment mode in “local” (−*T* = 0) for mining reads and in “overlap” (−*T* = 1) for contigs confirmation, gap existence cost -a = 1 and mismatch cost -q = *2* [18]. We converted the resulting *.maf* alignments to *.sam* using the “maf-convert” Python script. Host read-contaminants were removed after mapping against the human reference genome hg18. The filtered reads were mapped against the HCMV-TB40/E clone Lisa genome [9]. We selected this particular reference as it presented the highest similarity compared to our raw de novo assembled contigs. The resulting alignments were visualized with the *Integrated Genomics Viewer* (IGV). Crucially, we extracted the full sequences of those reads that had fragments longer than 500 bp aligned to the reference, based on their name and using in-house R scripts. It is of high importance to note that the LAST output (*.maf* file) contains only the aligned part of each read to the reference, thus the extraction directly from the *.bam* files, making use of the samtools flags, would result in partial sequences.

We performed the de novo assembly of the HCMV genome with Smartdenovo (https://github.com/ruanjue/smartdenovo) to generate extra-long contigs of relatively lower accuracy and with Spades [19] for the generation of highly accurate contigs but of shorter length, which were further merged using CAP3. We then retro-corrected the Smartdenovo contigs using the Spades contigs to generate a single full-length genome and several sub-genomic contigs (utg’s) which where merged again with CAP3 (ctg’s). Manual curation of misassemblies was performed by visual inspection after remapping the raw reads to the contigs and confirming the continuousness and the uniform depth of the alignment. We further curated the final assembly sequence using Pilon [20] in two rounds of remapping of the reads to the final contigs. All assemblies were evaluated with QUAST [21]. We filtered the QUAST-misaligned contigs with BLAST to exclude those suggesting rearrangements at the beginning or at the end of repetitive regions but not expanding into the unique regions. The remaining contigs were visually inspected and evaluated using MAUVE [22]. The annotation of the full genome construct was performed with RATT [23] based on the TB40E-Lisa reference strain.

We called SNPs and INDELs using *samtools mpileup* and *bcftools* [24], keeping variations supported by at least 80% read concordance and 5 reads depth per position, after direct comparison with the TB40/E reference strain. Using snpEff (v4.3 s) [25] the resulted .vcf files where annotated to the reference genome and SNPs were further filtered with snpSift (v4.3 s) [26]. We estimated the per-gene divergence, after dividing the total number of variations by the length of each protein. To calculate the mean coverage of the reads across the main de novo assembled genome, we used “*bedtools coverage*” [27]. The coverage plots in comparison to the GC content across the genome and the genomic synteny comparisons were visualised using Artemis [28].

We estimated the Neighbor-Joining consensus tree after aligning 29 representative full genome sequences and the de novo assembled genome using MAFFT v7 (https://mafft.cbrc.jp/alignment/software/) and the FFT-NS-2 algorithm.

## Results

### Hybrid de novo assembly of HCMV genome using only MinION data

**Fig. 1:**
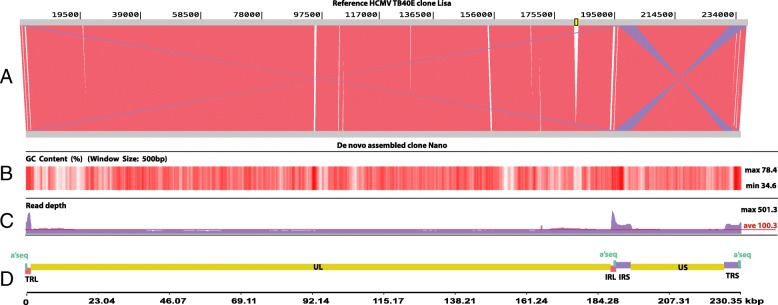
De novo assembled genome characteristics. **a**: De novo assembled TB40/E clone Nano aligned to TB40/E clone Lisa reference (top). Forward alignment blocks are in red and reverse in purple. The deleted region of UL144 and UL145 genes is in yellow. **b**: GC % heat-map, white regions correspond to lower (min 34.6%) and dark-red regions correspond to higher (max 78,4%) GC content. **c**: read-depth across the de-novo assembled construct (confirmative re-mapping of raw reads), red line represents the average depth (100.35). **d**: annotated map showing the basic genome segments

We used the MinION Nanopore sequencer, in combination with the compatible, transposase-based, library preparation kit to analyse a HCMV TB40/E polyclonal culture sample. Our starting material was expected to include mixed viral genomes as a result of multiple fibroblast passages. We developed a novel bioinformatics pipeline to reconstruct the viral genomes and reveal structural variations. The lengths of the raw reads (45,965 in total) ranged up to 365,569 bp; 26,497 (57.64%) reads of 6450 bp average length were mapped to the reference, while the largest was 56,310 bp long. The assembly was confirmed by remapping of the reads against it, resulting in an average read depth of 100.3 X. The coverage was uniform over the Unique regions but was increased over repeated genomic regions with higher GC content and lower complexity, like the a’ sequence, due to multiple, non specific alignments of shorter reads, lacking US or UL segments (Fig. 1). The visual inspection of the mapping alignment indicated that the reads were continuous and interlaced confirming the delineated genomic synteny.

**Fig. 2:**
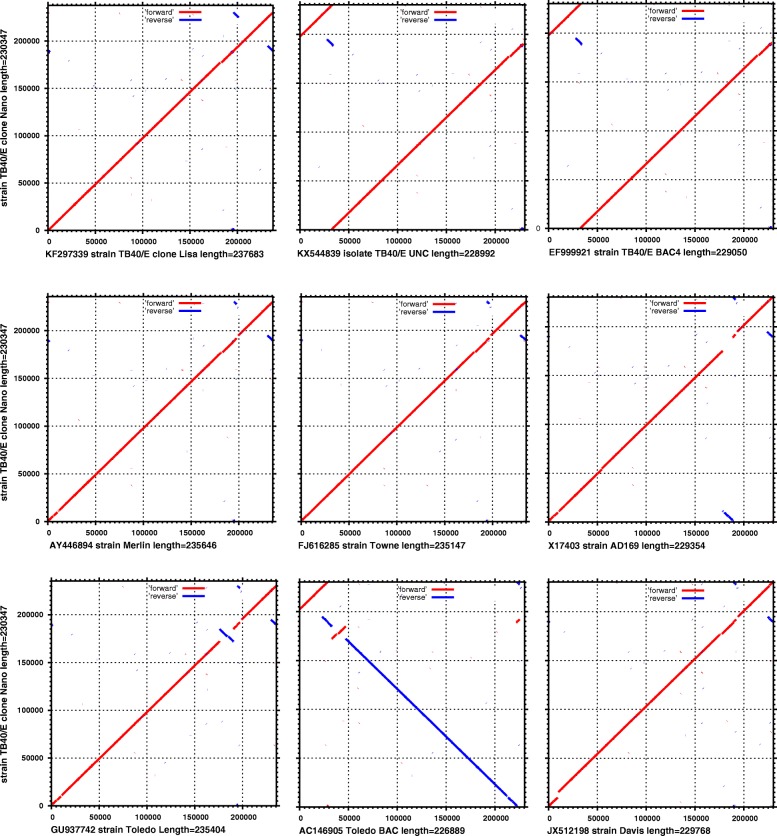
Genome-wide similarity comparison of the de novo assembled HCMV TB40/E genome (vertical) with nine representative HCMV strains (horizontal). Forward alignments are in blue, reverse alignments are in red

**Fig. 3:**
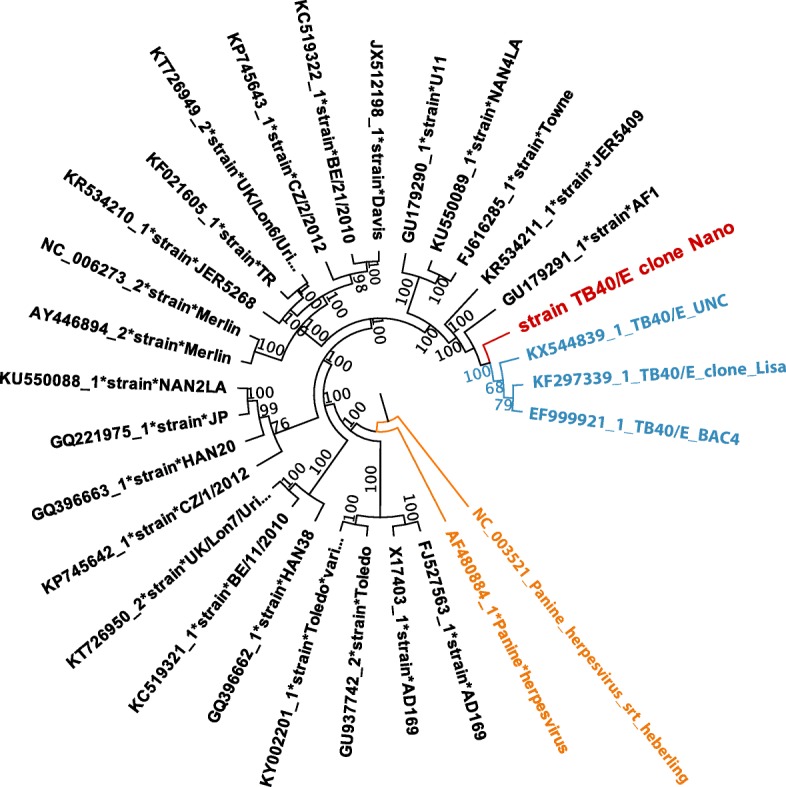
Full genome phylogenetic analysis of the de novo assembled clone Nano (in red) and 29 representative strains. Two Chimpanzee cytomegalovirus (Panine Herpesvirus) strains were used as tree roots (orange). HCMV TB40/E strains are shown in cyan

The hybrid bioinformatics algorithm dramatically improved the assembly compared to the solo use of the Spades assembler. In detail, the total number of contigs was reduced from 127 to 37, while the N50 was increased from 5689 to 41,056 and the covered genome fraction from 92.54 to 98.62% (Table 1). Moreover, our approach provided contigs of higher similarity to the reference (> 97% in blast alignments, data not shown) compared to the solo Smartdenovo assembled contigs, which presented only ~ 83–86% similarity to the reference. Thus, we show that reads produced from a single run of MinION were sufficient to assemble the HCMV TB40E genome in a single contig, without the need of scaffolding. The resulting sequence length (230,347 bp) is very close to the TB40E BAC clone (EF999921) and the isolate UNC (KX544839) but the structure of the genome was similar to the ~ 7000 bp longer clone Lisa (KF297339) (Fig. 2). Comparing the final sequence to a group of 29 representative full-genome unique HCMV sequences, we classified it within the TB40/E clade (Fig. 3).

**Table 1:** Comparison of de novo genome assembly methods

	Hybrid Assembly	Spades Assembly
Assembly vs. reference statistics		
Genome fraction (%)	98.62	92.54
Duplication ratio	3.85	1.60
Genes covered	170 + 0 partial	144 + 24 partial
Largest alignment	175,612	13,936
Total aligned length	900,508	350,687
NG50	230,347	9304
NG75	230,347	6488
Statistics without reference		
Contigs	37	127
Largest contig	230,347	17,689
Total length	985,159	452,509
N50	41,056	5686
N75	22,636	3766
GC (%)	57	57

### The effect of the hybrid assembly algorithm on deciphering structural variability and non-canonical contigs of HCMV genomes

**Fig. 4:**
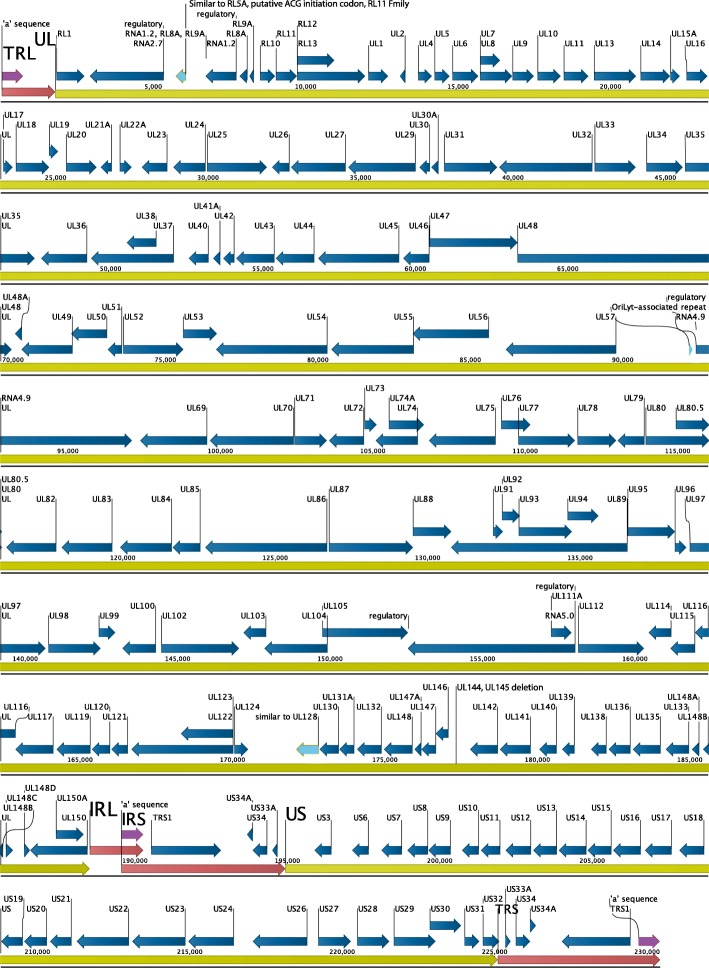
De novo assembled clone Nano annotation. Red arrows represent terminal and internal repeated sequences, green arrows represent UL and US regions, purple arrows indicate the 3 repeats of “a” sequence, blue arrows represent the annotated genes and light blue arrows correspond to miscellaneous features. UL 144 and UL 145 are missing due to a 1348 bp deletion at position 177,379 within UL region, which corresponds to coordinates 181,942–183,290 of clone Lisa

We closely examined the de novo assembled genome but also all the 36 alternative contigs, to identify structural variations. We observed a 1348 bp deletion within UL (181942–183,290 with respect to clone Lisa - KF297339) affecting the UL144 and UL145 genes. The deletion was supported by the main construct and all the alternative contigs (Fig. 1)**,** suggesting that the deletion is not a sequencing artefact. The low-complexity variable number tandem repeats (VNTRs) in the TRL and the IRL region showed as expected difference in the copy numbers both in the main construct as well as within alternative contigs when compared to the published sequence. We conclude that the assembled genome was complete, allowing the transfer of almost all the 172 genomic features from the reference strain, with the exception of UL144 and UL145 that were missing (Fig. 4).

### Detection of structural and point mutation quasi-species variants

**Fig. 5:**
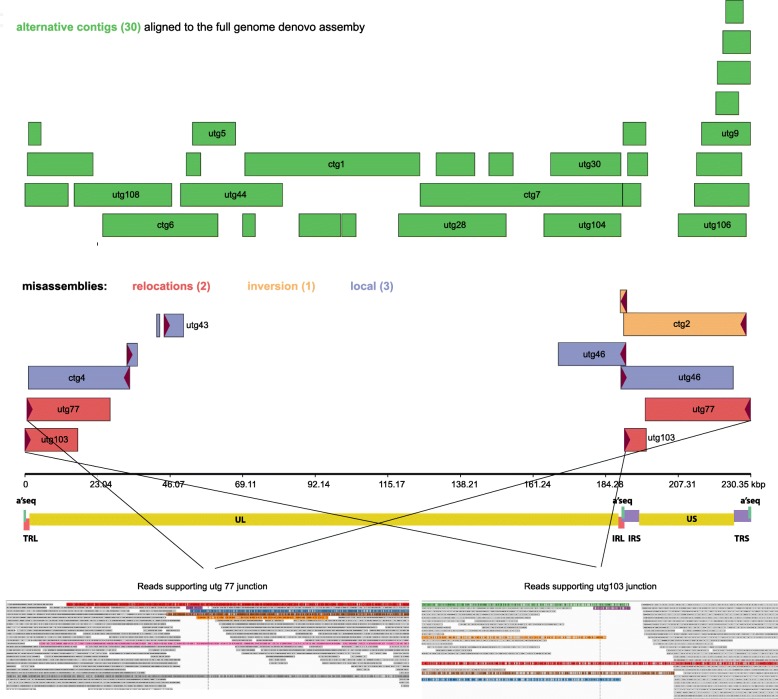
Structural variations of alternative contigs compared to the full genome construct. Green boxes represent sequence-divergent contigs sharing the same synteny with the full genome construct. Red (relocations) purple (local misassemblies) and orange (inversion) boxes represent misaligned contigs, which indicate the presence of viral quasi-species in the culture. Reads supporting some major rearrangements are colored, in the bottom IGV screenshots

We expected that the MinION sequencer would capture non-canonical HCMV genomes present in our culture. Indeed when we filtered and visually confirmed misaligned contigs (those that are not aligned in a canonical way to the reference during the assembly evaluation with QUAST), we found one inversion (ctg2), two relocation events (utg77 and utg103) and three locally misaligned contigs (utg43, ctg4 and utg46) suggesting indels within the UL and the IRL regions. The assembly approach further produced 31 alternative contigs, with the same phasing but their sequence was variable in composition compared to the main construct. These alternative contigs were dispersed across the genome resulting in 3.85X duplication ratio (Table 1), but the phenomenon was more intense over the US –TRS region of the genome (Fig. 5).

**Fig. 6:**
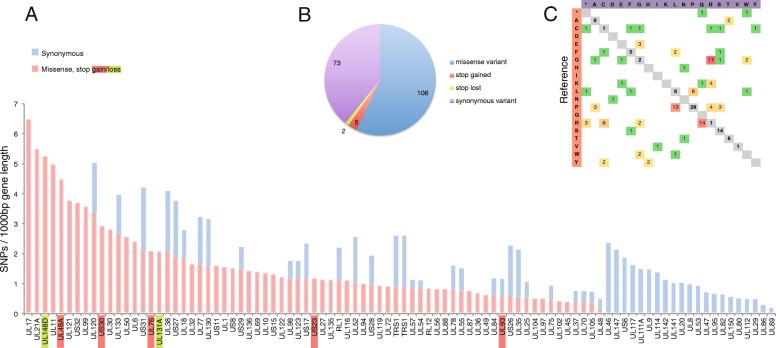
Genome-wide mutation analysis. **a** Gene-length-normalized SNPs rate. Non-synonymous mutations (missence, stop gains, stop-losses) are shown in pink, while synonymous mutations are in cyan bars. **b** Total synonymous / non-synonymous SNP distribution. **c** Cumulative Amino-acid changes (heat-map). Reference amino-acids are shown in the vertical axis. The synonymous mutations are distributed in the grey diagonal

We estimated the average divergence per gene across the HCMV reference genome, after dividing the total number of SNPs detected for each gene by the respective gene length. We manually characterized the UL11 mutations (genomic location 19,450–19,700 bp) as it comprises a low complexity region which thus attracts a non-specific pile of reads (> 6,000X coverage) and many inaccurate SNP calls. We further identified 73 synonymous and 80 missense SNPs, out of which 5 were stop codon gains (UL48A, UL76, UL93, US23, US30) and 2 where stop codon losses (UL148D, UL 131A). A group of 21 genes presented only synonymous variants in their sequences. Analyzing the substitution rate of the aminoacids we observed a skew towards 3 particular changes (P➔L, R➔Q, and G➔R) (Fig. 6).

## Discussion

The de novo assembly of HCMV and other herpesviruses genomes, is challenging due to the increased length and its unique structure, which is characterized by extended, repeated, internal and terminal regions, but also by omnipresent low-complexity sequences that usually exceed the read-length of currently available next generation sequencing platforms [29–31]. Full genome de novo assembly of HCMV will be useful in understanding the full extent of the intra- and inter-host genomic variability but also the variability that results from selective pressure e.g. antiviral therapy.

To date, the analysis of the HCMV genome has been only based on 2nd generation sequencing platforms that deliver short-read HTS lengths (reviewed in [32]). As a result, the assembly of the virus has been based either on solo mapping alignments [12] or on hybrid approaches, like the construction of the consensus genomic sequences from de novo assembled contigs supplemented with parts of the reference sequence to fill in the assembly gaps [13]. The MinION sequencer has already been used to improve the de novo assemblies of data generated by Illumina HiSeq platforms [16], while we have also shown that MinION can improve de novo assemblies of HHV-1 derived from the Roche 454 GS Junior sequencer [17]. In this study, we developed a novel bioinformatics pipeline, in order to explore the potential of the MinION nanopore sequencer to de novo reconstruct the full HCMV genome, without using supplementary reads from other platforms. Our intention was not to use a reference sequence to fill gaps in the assembly or to guide the contigs scaffolding, thus introducing synteny bias, since it is known that the virus is highly polymorphic both in culture and in the host [33]. Indicatively, two of the three currently available TB40/E sequences do not share the same genomic structure (Fig. 2).

Indeed, we were able to reconstruct the HCMV genome in a single contig, achieving uniform and continuous read coverage across this construct in the confirmatory remapping. Crucially, this was made feasible due to the implementation of our hybrid algorithm, which results in longer contigs, of high accuracy (Table 1) and provides a model method for the optimum usage of long-read data for challenging tasks as the de novo assembly of large and highly repetitive viral genomes. The assembled sequence was correctly classified within the TB40/E clade in our confirmatory phylogenetic analysis (Fig. 3), providing a proof that MinION can be used as a versatile, alternative platform for the molecular surveillance of HCMV and for full genome epidemiological studies, as for example in cases of smaller viruses like Ebola [34] and Zika [35].

Apart from the assembling limitations characterizing other short-read platforms, the respective library preparation protocols involve PCR amplifications, which have been shown to introduce artificial recombination events in the highly repetitive context of the HCMV genome [14]. Here, we present a PCR-free approach for the rapid library preparation and sequencing of HCMV with the MinION sequencer. This approach is not prone to artificial recombinants and, given the extra long reads produced by the nanopore technology, provides a unique combination for the structural analysis of the HCMV genome. Consequently, we were able to accurately reconstruct not only the full genome of the virus, but also to capture overlapping contigs of alternative sequences, and, most importantly, contigs suggesting rearrangement events. These rearrangements have occurred between the major segments of the genome, with the repetitive sequences to serve as recombination hot-spots and were supported by long reads running through the repetitive and expanding into the unique regions. Although our data did not support the full genome reconstruction of these recombinants, they provide evidence of the existence of isomerized quasi-species’ genomes in our cultures (Fig. 5). Using the method of molecular combing, other groups have described structural variants such as isomers, rearrangements and non-canonical genomes in HHV-1 cultures [36]. Just like HHV-1 [37], HCMV’s class-E genome is also known to create isomers [2] and here, we prove for the first time that MinION captures such structures in a much simpler way compared to complex molecular techniques. Our reference-free approach allowed mining of minority reads that correspond to structural variants. For example, a handful of reads support the isomers found in our analysis, which otherwise - using the reference sequence to guide the assembly- would have been ignored.

The GC content across other Herpesviruses genomes has been shown to reduce the read depth in mapping assemblies [38] mainly in the inverted repeats (I/T-R-L/S), which incorporate numerous VNTRs. The latter also disrupt the de novo assemblies due to inter-sample variation and generation of conflicting contigs [29]. The initial mapping of our raw reads on the reference HCMV Merlin or TB40/E-Lisa were in accordance with these observations, with multiple gaps present over the repeated regions (data not shown). In contrast, our confirmatory mapping of the raw reads on the de novo assembled genome showed the opposite trend, that is an increased read depth across the repeated sequences, due to duplicated mapping of shorter reads, lacking unique sequence segments. The depth was more than double compared to the rest of the genome (a’-TRL: 372.7X, IRL-a-IRS: 246.4X, TRS-a’: 206.9X), which is in accordance with the aneuploidy of the respective sequences in the genome. At the same time, we observed multiple copy number variation of the same VNTRs in the alternative contigs of our assembly. Our results support the hypothesis that the gaps in the mapping alignments are not due to the GC content and are mainly driven by discordances of the sequenced sample with the reference and most probably with the VNTR copy numbers. Numerous studies provide evidence that the variations of the VNTRs are linked with the functionality and the pathogenicity of specific strains of viruses [39–43], while they can be also used for the epidemiological identification of clinical isolates [44, 45]. MinION can unambiguously resolve these loci, due to the increased length of its reads and can provide information regarding the clonal diversity of the polymorphic quasi-species present in the sample. Our results suggest that future studies focusing on the resolution of the clinical and epidemiological aspects of virus VNTRs should make use of longer reads derived from 3rd generation sequencers like MinION.

Nucleotide differences are dispersed throughout the HCMV genome, but the genes are not equally conserved. Comparing our sample with the TB40/E clone Lisa reference sequence, we found substitutions in the UL1, UL6, UL8, UL9, UL10, UL11 and UL147 genes, which are members of the RL11 (RL11–13, UL1 and UL4–11) and the CXCL (UL146, UL 147) gene families respectively. These findings agree with a recent, high-resolution study of the HCMV inter-host diversity, which revealed that the virus is more divergent than other Herpesviruses, highlighting these particular gene families as hot spots of higher genomic diversity [46].

We have identified a 1348 bp deletion affecting UL144 and UL145 genes. UL 144 (truncated tumor necrosis factor receptor) activates NF-kB in a TRAF6-dependent manner which in turn upregulates the chemokine CCL22 (MDC) [47] and inhibits T cell proliferation [48]. Mutations in this gene have been related with the clinical outcome of congenitally infected infants and with the viral loads [49–51], however, others found no evidence of these correlations [52]. UL145 may act as an intra-nuclear regulating factor binding directly to the host DNA and is predicted to contain 1 protein kinase C and 2 casein kinase II phosphorylation sites and also a zinc finger structure and is generally conserved in clinical isolates [53]. Like Towne varS and AD169 variants, strain TB40/E is also mutated in UL/b’, and there is at least one derivative (TB40/E-Bart) additionally lacking UL145 and UL144 [5, 9], like our sample. Cells infected with TB40/E-Bart were more sensitive to NK cell–mediated lysis, compared to those infected with strain TB40/E-Lisa or Toledo. While our TB40/E culture was expected to comprise mixed viral quasispecies, the delta-144/145 population was dominant in our particular preparation.

## Conclusions

Although the HCMV de novo assembly is challenging, our bioinformatics pipeline in combination with the increased accuracy of the latest versions of MinION allowed the complete assembly of the HCMV genome and revealed major genomic rearrangement events. The genomic material used for the library preparation in this study was extracted from virus cultures, thus was of high purity and concentration. In the case of clinical samples however, the viral DNA typically represents only a small fraction of the total genomic material. Additional enrichment strategies based on biotynilated baits might have to be employed in such cases, as they efficiently increase the proportion of viral reads and improve the assembly of the virus genome [54–56]. Our study supports the theoretical prediction that long-read technologies can boost the generation of accurate viral-genome assemblies especially in viruses with large genomes, a development of higher importance in defining genetic determinants of drug resistance, virulence, pathogenesis and viral evolution.

## Abbreviations

cpe: Cytopathic effect
DAPI: 4,6-diamidino-2-phenylindole
DMEM: Dulbecco’s Modified Eagle Medium
FCS: Fetal calf serum
HCMV: Human cytomegalovirus
HFF: Human foreskin fibroblasts
HHV1: Human Herpes Virus 1
HTS: High-throughput sequencing
IGV: Integrated genomics viewer
INDELs: Insertions and deletions
MAP: MinION early access program
ONT: Oxford nanopore technologies
PBS: Phosphate Buffered Saline
RNN: Recurrent neural networks
SNPs: Single nucleotide polymorphisms
TLR/ILR: Terminal and Internal Long Repeated sequences
TSR/ISR: Terminal and Internal Short Repeated sequences
UL: Unique long
US: Unique short
VNTRs: Variable number tandem repeats
